# Dose Assessment in Mining Communities in South Africa Using Ecolego Simulation Software

**DOI:** 10.3390/ijerph23010003

**Published:** 2025-12-19

**Authors:** Violet Patricia Dudu, Masengo Ilunga, Dunisani Thomas Chabalala, Manny Mathuthu

**Affiliations:** 1 Department of Civil & Environmental Engineering and Building Science, University of South Africa, (Florida Campus), Private Bag X6 Florida, Roodepoort 1710, South Africa; ilungm@unisa.ac.za (M.I.); chabadt@unisa.ac.za (D.T.C.); 2 Centre for Applied Radiation, Science and Technology (CARST), North-West University (Mafikeng Campus), Private Bag X2046, Mmabatho 2735, South Africa; manny.mathuthu@nwu.ac.za

**Keywords:** Ecolego, modeling, dose assessment, inhalation, exposure, radionuclide

## Abstract

Ecolego simulation was used to predict long-term behavior of radionuclide concentration in air and soil and to estimate health impacts. The radionuclides considered in this model are ^238^U, ^232^Th, ^226^Ra, ^210^Pb, ^210^Po, ^234^Th, ^230Th^, and ^234^U. Dose assessments were conducted in soil and the pathway to humans through inhalation for an adult and an infant (1–2 years). Model simulations were performed over a period of 100 years, the approximate human lifetime. The doses through inhalation to adults were higher than the doses to infants in all study areas A to C. The inhalation doses for an infant in the three study areas A, B, and C range from 1.86 × 10^−2^ µSv to 43.45 µSv, whereas those for an adult vary from 3.58 × 10^−2^ µSv to 271.56 µSv. The total dose from inhalation of the eight radionuclides for an adult varies from 1811 µSv/y in area C to 2015 µSv/y in areas A and B, yet that for an infant was 744.51 µSv/y in area C and 745 µSv/y in areas A and B. These results will assist in developing more effective strategies for monitoring and mitigating exposure risks and pave the way for enhanced regulatory policies aimed at safeguarding public health and the environment.

## 1. Introduction

While radionuclides occur naturally in soils and rocks as a result of radioactive decay, most environmental releases are due to industrial processes such as uranium mining. Mining and processing of uranium have negative impacts on the environment, including contamination of the environment by radionuclides and toxic components. Some mining activities involve minerals that contain low levels of radioactive isotopes that become concentrated in mine tailings [1]. These isotopes can be released to the environment as dust through drilling, blasting, overburden loading and unloading, and transport materials. The transport of these radionuclides in the environment is enhanced when the dust gets blown off the mining sites by wind. Thus, mining activities lead to increases in concentrations of particulate air pollutants that reduce the quality of air in the atmosphere. Epidemiological studies have shown that people living near mine dumps are at risk of exposure to different pollutants via different pathways [2,3]. This is especially dangerous where tailings generated can end up contaminating the environment, resulting in the exposure of different organisms due to uptake in the food chain [4,5]. The routes for uptake of metals and radionuclides are through ingestion and inhalation [5,6]. The adverse effects of radionuclides in the human body emanate from the deposition of energy in the tissues because of radioactive decay. Hence, ionizing radiation is a well-known risk factor for human cancer [7,8]. Carcinogens are considered to have no dose below which adverse effects occur. In other words, they have no threshold.

Ecolego simulation can be used for conducting risk assessments of complex dynamic systems evolving over time in the field of radiology. It is of much importance to predict the long-term behavior of radionuclide concentration in air and soil and to estimate its health impacts. It consists of blocks that interact together to produce the model. Examples of blocks include compartments, delay, expression, sink, source, transfer, and influences [9]. The pathways and processes by which radionuclides can move in the environment can be very complex and can vary with the location. Hence, for maximum efficiency, a model should have the smallest number of compartments and pathways for the dynamics to be realistic [10]. Pathways for radionuclide penetration into the human body include ingestion of contaminated water and food and inhalation of contaminated air. A radionuclide enters the system from a source such as mine tailings via deposition onto the surface soil. The radionuclide is transferred from the surface soil to the atmosphere through resuspension, and the radionuclide can also be deposited from the air onto the soil.

When considering exposure via inhalation, this can be two-fold: indoors and outdoors. However, because of the shielding effect of buildings, indoor exposures may be lower than outdoors [11]. Thus, this study assumes exposure time of one hundred percent from outdoors, which gives a conservative estimate since the radionuclide contamination of air comes from resuspension of soil particles. The 100% outdoor exposure is a conservative default value used to ensure that the calculated dose does not underestimate the risk when assessing long-term exposure to radionuclides and is used in worst-case scenarios to ensure public protection. Interaction of radionuclides with different mobile carriers in air and exchange processes can be complex, as there are many factors that can be considered. Some factors include radionuclide properties, weather conditions (transport velocities of contaminants and carriers, temperature), spatial concentration of radionuclide, diffusion coefficient, and rate of migration of contaminants [10,12,13,14]. However, this study is a simple attempt based on the assumption that there is negligible exchange of contaminants between different carrier phases.

**Figure 1:**
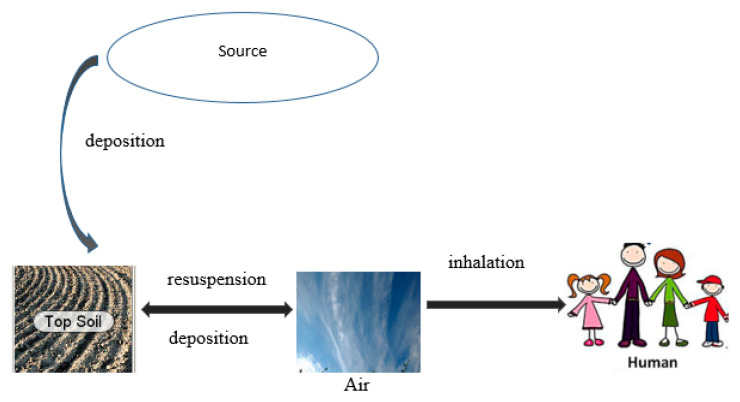
Conceptual model with pathway and processes of radionuclide releases.

Figure 1 shows the schematic representation of the interaction between the model and compartments as a flow diagram, and Table 1 shows the same information as an interaction matrix. The arrows and interactions labeled transfer represent fluxes of radionuclides to and from the compartments.

The aim of the study was to apply Ecolego simulation software Version 6.5 by Facilia, Sweden, in simulating dose assessment in humans. Specific objectives included estimating cancer incidence in mining communities in the study areas in South Africa using the software and focusing on the inhalation pathway as a route of transmission of radionuclides from dust.

Dose assessment using tools like Ecolego simulation software is important for identifying exposure pathways and quantifying the exposure doses so that at-risk populations can be determined. Such information is valuable for informing health promotion strategies to mitigate the risks and address goal number 3 of the Sustainable Development Goals, which is aimed at promoting good health and well-being. This study assesses contaminant doses in mining communities to provide critical data for health promotion strategies, such as community education, environmental remediation, or policy advocacy. This, in turn, can support policy changes to protect mining communities and enhance community well-being.

## 2. Materials and Methods

### 2.1. Description of the Study Areas

Three study areas, Mine A, Mine B, and Mine C, were selected. Mine A is in the Free State area, where gold and uranium mining are performed, whereas Mines B and C are located in the West Rand area (Carletonville). Both Mines B and C are involved with gold mining. In the Free State province, the area is characterized by a continental climate with warm to hot summers and cool to cold winters. The climate in the West Rand area (Highveld) has short, cold winters and long, hot summers with an average annual temperature of 16 °C. Precipitation mainly falls in summer, with the highest rainfall recorded in the month of January. Rainfall ranges from 400 mm to 600 mm per annum, with the lowest rainfall recorded in July [15,16].

### 2.2. Input Data

Ecolego Simulation Software Version 6.5 (Facilia, Stockholm, Sweden) was used for simulations. The dose assessment model facilitates dynamic compartment modeling of radionuclide transport and exposure pathways. Ecolego simulation software consists of linked compartments that represent environmental media such as soil, dust, or air, and human exposure routes. The focus is specifically on the inhalation of resuspended dust particles. Deterministic simulations used site-specific activity concentration data from field samples. The assumptions factored in this study are 100% outdoor exposure, uniform radionuclide concentrations in each compartment, steady dust resuspension rates in the dry season, age-specific breathing rates, and exposure times for adults and infants. The ingestion pathway has been excluded due to unreliable consumption data and unavailable transfer factors for the populations in the study areas. Some radionuclide-specific data, such as decay half-lives, decay constants, dose conversion factors, dose coefficients, leaching, soil density, and deposition rate values, have been derived from the literature. Documents from the International Commission on Radiological Protection, as shown in Appendix A, have also been used. Human-specific data, such as breathing rate and classification using age group, also follows the guidelines referenced [17]. In this model, only two groups are considered: an adult and an infant (1–2 years). Other parameters, including concentration in air, concentration in soil, and dust concentration, included in the model have been derived from experiments conducted in this study. Exposure factors, including exposure time, have been calculated, and the assumption is that an individual is exposed throughout the year, and these are members of the public. The model simulations have been performed over a period of 100 years, since this time period is taken as the approximate human lifetime. The next section focuses on outputs from the model, and only those relevant to the study have been considered.

### 2.3. Doses from Inhalation

The internal dose from inhalation of atmospheric air $D_{inh}$ (Sv/y) can be calculated using the equation:

(1) $$D_{inh}=C_{air}\cdot R_{inh}\cdot {DF}_{inh}$$

where $C_{air}$ is the concentration of the radionuclide in air (Bq/m^3^); $R_{inh}$ is the inhalation rate (m^3^/y) (default inhalation rates for adults and for 1–2-year-old infants are 8400 and 1400 m^3^/y, respectively); and ${DF}_{inh}$ is the inhalation dose coefficient (Sv/Bq) (values used are given in the International Commission on Radiological Protection (ICRP publication 60) [17,18]. For the effective dose coefficient, the activity median aerodynamic diameter (AMAD) was assumed to be 1 µm as recommended by ICRP when considering environmental exposures in the absence of specific information on the physical characteristics of the aerosol. The values for dose conversion coefficients used in the study are shown in Appendix A, Table A2.

### 2.4. Doses from External Exposure

The main contributions to external exposure come from gamma-emitting nuclides in the ^40^K, ^238^U, and ^232^Th decay series in soil [14].

The formula for calculating external dose $D_{ext}$ (Sv/y) is given as

(2) $$D_{ext}=C_{soil}{\cdot \rho }_{soil}\cdot H\cdot {dosCoef}_{ext}$$

where $C_{soil}$ is the concentration of the radionuclide in soil (Bq/KgDW), $\rho_{soil}$ is the soil density (kgDW/m^3^), H is the exposure time to external radiation (h/y) which is given a value of 8760 and ${dosCoef}_{ext}$ is the dose coefficient for external exposure of the radionuclide (Sv/h per Bq/m^3^) [17].

The dose coefficient of external exposure is defined as the dose rate to which an individual is exposed from unit volumetric concentration in soil of the radionuclide [11,19]. The recommended values are shown in Appendix A, Table A4. The values were derived from calculations for a silt soil with a density of 1600 kg/m^3^, 20% air, and 30% water, and taking into account the values for tissue weighting factors as recommended [17].

### 2.5. Calculation of Total Dose

The equation used in Ecolego simulation for calculating total dose ${Dose}_{Total}$ (Sv/y) is

(3) $${Dose}_{Total}={Dose}_{ext,\;Total}+{Dose}_{inh,\;Total}$$

### 2.6. Concentration in Air

The formula for calculating concentration in air ${Conc}_{air}$ (Bq/m^3^) is given by

(4) $${Conc}_{air}={Conc}_{soil}\cdot {Conc}_{dust}$$

where ${Conc}_{soil}$ is the concentration of the radionuclide in soil (Bq/kg) and, ${Conc}_{dust}$ is the concentration of the radionuclide in dust (kg/m^3^).

## 3. Results

The input parameters used to simulate radionuclide transfer from soil to air and to estimate exposure of the populations in mining areas to ionization radiation are shown in Appendix A. Simulations were performed and viewed using MATLAB graphical capabilities and exported to Microsoft Excel. Dose calculations have been carried out for the radionuclides: ^238^U, ^232^Th, ^226^Ra, ^230^Th, ^234^U, ^210^Pb, ^210^Po, and ^234^Th.

### 3.1. Inhalation Doses

The doses through inhalation to the adults were higher (~3 times) than the doses to infants aged 1–2 years in all study areas. Inhalation doses for an infant in the three study areas, Mine A, Mine B, and Mine C, range from 0.0186 µSv/y to 43.45 µSv/y, whereas those for an adult vary from 0.0358 µSv/y to 271.56 µSv/y. For an infant, the highest contribution to dose was due to inhalation from ^234^Th (43.45 µSv/y), and the least contributor was ^210^Po (0.0186 µSv/y), followed by ^210^Pb (0.024 µSv/y). For an adult, ^234^Th had the highest contribution to dose (271.56 µSv/y) due to inhalation, followed by ^232^Th (109.50 µSv/y), and the least doses were contributed by ^210^Po (0.0358 µSv/y), followed by ^210^Pb (0.0466 µSv/y). Table 2 shows the inhalation doses for an adult and an infant.

For most radionuclides, the doses are the same for both adults and infants across the mining areas. The exception is ^234^Th in Mine A for adults with a lower dose than Mines B and C, showing the specific area differences. The higher doses that adults receive compared with infants for all radionuclides except ^234^Th could be due to the age-specific default values and physiological differences assumed in the model, where adults have higher breathing rates and longer exposure times as compared with infants.

### 3.2. External Doses

#### Total External Dose

**Figure 2:**
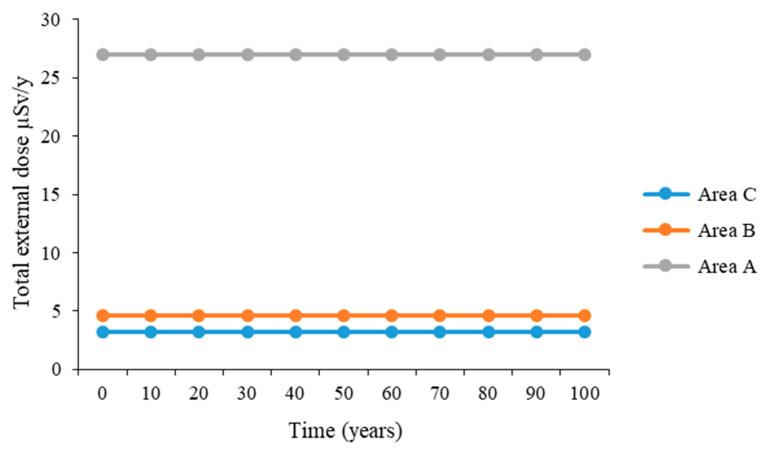
Total external dose to an adult from radionuclides in the study areas.

The total external dose to an adult from the radionuclides in area A was the highest, with a value of 27 µSv/y, followed by that in area B, with a value of 4.63 µSv/y, and that in area C was the lowest, with 3.17 µSv/y, which was almost nine times lower than that in area A. Figure 2 shows the results.

**Figure 3:**
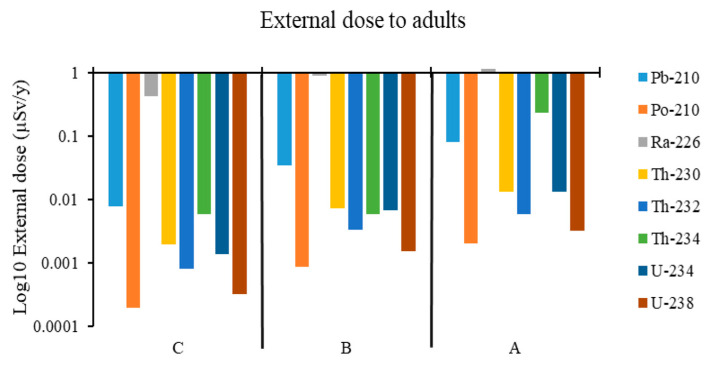
Log10 external dose from radionuclides for adults in areas A, B, C.

The external doses from the different radionuclides were found to vary a lot from the study areas, as shown in Figure 3. In the mines A, B, and C, ^226^Ra contributed a larger proportion, with the least external dose from ^226^Ra being 0.42 µSv/y in Mine C, 0.89 µSv/y in Mine B, and 1.15 µSv/y in Mine A.

**Figure 4:**
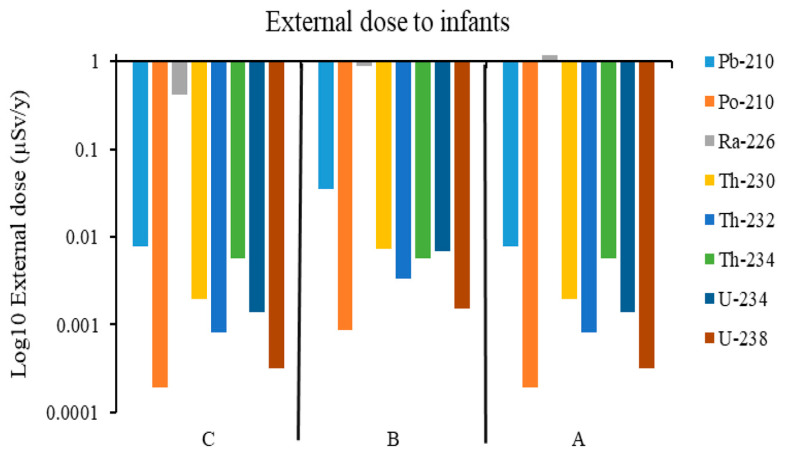
Log10 external dose from radionuclides for infants in areas A, B, C.

A similar trend was observed in external doses for infants, with the main contribution to the external dose from Ra-226. Figure 4 shows the external dose from the different radionuclides for infants.

**Figure 5:**
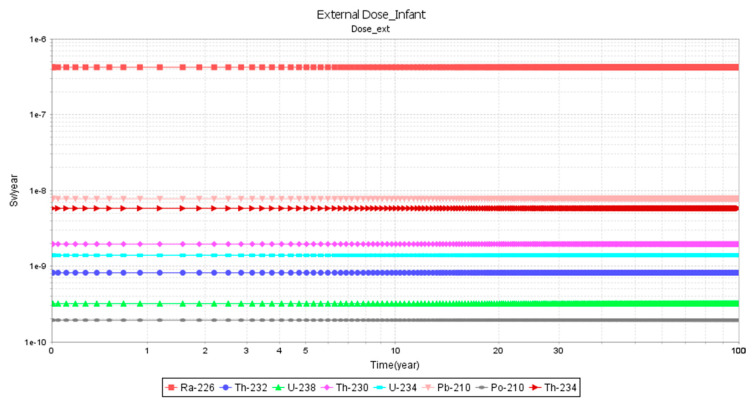
External dose for an infant in area C.

**Figure 6:**
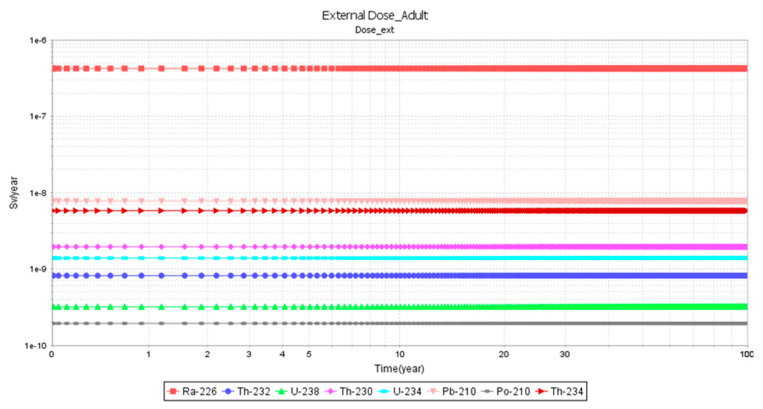
External dose for an adult in area C.

Figure 5 and Figure 6 show the graphical simulations of external dose in study area C for an infant and an adult, respectively, for a time period of 0–100 years. For both infants and adults, there were no differences in the external doses for Ra-226.

### 3.3. Total Dose

**Figure 7:**
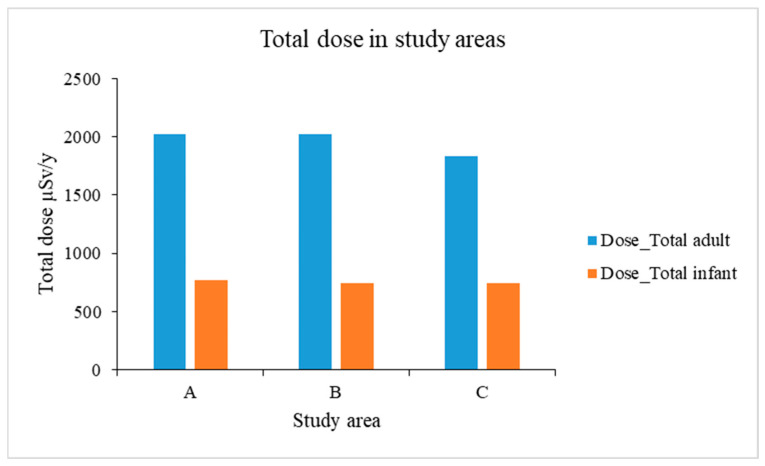
Total dose in the study areas for infants and adults.

The collective total dose was estimated to be 2020 µSv/y for an adult in area A, 2019 µSv/y in area B, and 1838 µSv/y in area C for an adult. For an infant, the total doses obtained were 772 µSv/y, 749 µSv/y, and 748 µSv/y for the areas A, B, and C, respectively, as shown in Figure 7.

### 3.4. Concentration of the Radionuclides in Air

**Figure 8:**
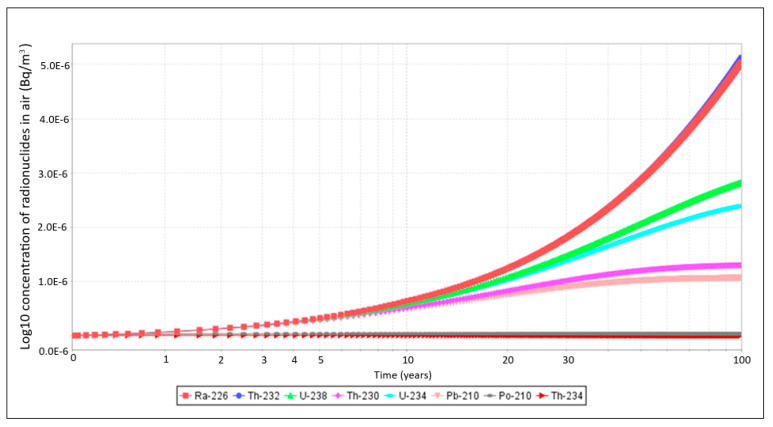
Log10 concentration of radionuclides in air over time in Mine B.

Simulations were also carried out based on Equation 4 to evaluate the concentration of the different radionuclides over a time period of 100 years. This was performed because, in the conceptual model, it is shown that radionuclides in the air can enter humans through the inhalation pathway. The graph of the log10 concentration of radionuclides in air over time in Mine B is shown in Figure 8.

## 4. Discussion

### 4.1. Trends in Radionuclide Concentrations

#### 4.1.1. Inhalation Doses

From the simulations performed, the inhalation dose for each radionuclide stays constant for the next 100 years. This is because some radionuclides, such as the uranium series isotopes, reach secular equilibrium, causing only gradual changes unless the source term changes. As expected from the literature, the ^226^Ra activity concentration is greater than that of ^238^U. ^226^Ra is a decay product of ^238^U in the uranium decay series with a shorter half-life (1600 years) than its parent (4.5 billion years) [20,21].

Mining can disturb the state of equilibrium between supply and decay, leading to increased concentrations of ^226^Ra. Since the three study sites are involved in uranium mining, ^226^Ra can accumulate in soils, leading to high concentrations of ^226^Ra. For the ^238^U series, ^226^Ra is responsible for the main contribution to inhalation dose. Other radionuclides could be neglected if the activity concentrations are of the same magnitude. The average annual effective dose from inhalation of uranium and thorium series (with the exception of radon and thoron) is between 5 and 10 µSv/year [22]. For the intake of long-lived radionuclides, the contribution to the dose from the parent is assumed to be very significant compared with the dose from short-lived daughter products. The short-lived daughter products are those with a half-life of days or shorter in the environment, and these are assumed to have a negligible contribution to the dose. However, after intake of parent radionuclide, the production and decay of short-lived daughters in the body may contribute significantly to the dose [23]. ^222^Rn is a natural radioactive gas produced by the decay of radium (^226^Ra), which in turn is derived from the radioactive decay of ^238^U. It has a half-life of 3.82 days and provides 50% of the total radiation dose to an average person [24,25]. It is estimated that every year in the United States, there are 168 cancer deaths, 89% from lung cancer caused by breathing radon released from water, and 11% from stomach cancer caused by drinking water containing radon [24]. It is often assumed that airborne radon poses a much greater health risk than ingested radon and that, looking at the effective dose equivalent for the whole body, radon yields an index damage of two to three orders of magnitude compared with all other radionuclides [25,26].

Inhalation doses differ for different radionuclides. Dose coefficients for inhalation of radionuclides include all contributions to the dose from the growth of daughters in the body [17,23]. This is because after intake of the parent radionuclide, the production and decay of short-lived daughters in the body may contribute significantly to the dose.

#### 4.1.2. External Doses

In terms of the total external dose from the radionuclides, mine A was found to have the highest with a value of 27 µSv/y, followed by that in Mine B with a value of 4.63 µSv/y, and that in Mine C was the lowest with 3.17 µSv/y, which was almost nine times lower than that in Mine A (Figure 2). The total external dose in Mine B was higher than that in Mine C by a factor of 1.5. The explanation for the trend is that Mine A had the highest activity concentrations of the radionuclides in soil, resulting in the corresponding high external doses to both adults and infants. Mine C had the least activity concentrations of the radionuclides in soil, resulting in a corresponding lower external dose to the adults and the infants in that environment. This is because in the model, the derivation of external dose considers the soil density, exposure time, dose coefficient conversion factor, and the radionuclide concentration in soil, of which the three parameters are constants except for the radionuclide concentration in soil. The highest doses are expected when humans spend time in areas with the highest radionuclide concentrations, drink water, and eat food that comes from these areas. The external doses varied a lot in all study areas, and a similar trend was observed for both adults and infants, with the main contribution to the external dose from ^226^Ra. About 98% of the external dose from the ^238^U series is delivered by the ^226^Ra sub-series [27]. This supports the results in this study, which show that ^226^Ra has the greatest contribution of all the radionuclides to the external dose. The radionuclide ^234^Th contributed a significant proportion to the external dose for adults with a value of 0.23 µSv/y in Mine A, whereas the contribution in Mines B and C was only 0.00577 µSv/y. The contribution from ^210^Pb was 0.08 µSv/y in Mine A, 0.035 µSv/y in Mine B, and 0.00772 µSv/y in Mine C. Contributions from other radionuclides were very small, as can be seen in Figure 4. The trend in increasing order of the study areas from external dose on infant to Ra-226 was C > B > A, though the same values as in adults’ external dose were recorded. The reason is that the external dose coefficients used for the radionuclides are the same. However, the exposure time used for the adults and infants is different, so this shows that infants are at a high risk of radiation exposure to the external dose. Radiation risks in children are expected to be higher because of the many cells that will be dividing in younger individuals and also because of the longer lifespan available for a potential cancer to be expressed [28,29]. ICRP uses dose coefficients that consider age-dependent biological effects. Infants are very vulnerable to radiation risks due to their heightened biological sensitivity and longer latency periods for potential health problems caused by radiation [6,17]. The Th-234 value in areas B and C was 40 times less than that in area A, showing that populations in A are more exposed to the external dose from ^234^Th than those in areas B and C. The modeled results in this study are well below the public dose limit recommended by ICRP and enforced by the National Nuclear Regulator (NNR) of South Africa. The elevated concentration of Th-234 in Mine A underscores the importance of site-specific dose evaluations and long-term monitoring strategies.

#### 4.1.3. Total Dose

The total dose due to inhalation in Mine C is lower than the world average of 1256 µSv/y, yet Mine B and Mine A surpassed the world average. However, there are some places that have reported higher values of inhalation doses than the ones obtained in this study, for example, in Canada, values as high as 3225 µSv/y were recorded, though ^222^Rn was the main contributor to the dose [30]. The reason for ^234^Th having the highest contribution was that the ^234^Th concentration in air was set at a higher value (default value of 1.0 Bq/m^3^) than other radionuclides, hence this is expected. Another possible reason for the high doses of ^234^Th is that it is a product of the ^238^U decay series with a half-life of 24.1 days, and it decays by emitting alpha particles. This means that secular equilibrium is attained in a period of less than 240 days [21]. Even if Mine C recorded total doses due to inhalation below the world average of 1256 µSv/y, it should be known that there is no safe dose for radiation, although it is well known that the higher the dose, the higher the risk of damage.

The collective total dose (summation of inhalation and external doses) has been estimated to be 2020 µSv/y for an adult in Mine A, 2019 µSv/y in Mine B, and 1838 µSv/y in Mine C (Figure 7). For an infant, the total doses obtained were 772 µSv/y, 749 µSv/y, and 748 µSv/y for Mines A, B, and C, respectively. Although the radionuclide concentrations in the mines differ, which contribute to the external dose, the total dose shows limited variability for the same age groups in the three mines. The total dose in both adults and infants due to external exposure in increasing order was C < B < A. The reason for limited variability in total dose of the same age groups could be that during the simulation, one assumption made was keeping constant values for the radionuclide concentrations in air, hence the contribution from the soil mainly affected the external dose results. Thus, the contribution due to inhalation was constant in Mines A, B, and C, except for the external dose. Otherwise, if soil ingestion was considered as another pathway in the model, then this could have altered the results, and higher values for the total dose could have been obtained. However, since this research did not focus on ingestion, the soil ingestion pathway was not included in the model.

### 4.2. Concentration of Radionuclides in Air

Figure 8 shows that the concentrations of radionuclides in the air remain fairly constant up to 1 year and then decrease for ^234^Th and ^210^Po during the time period until the next 100 years. For ^226^Ra and ^232^Th, there is an increase that is steady and steep from 30 years until 100 years. ^238^U and ^234^U concentrations increase with time but at a slower rate than ^226^Ra and ^232^Th. This may suggest that during their release into the air, the processes governing their decay or dispersal are less efficient compared with those of ^226^Ra and ^232^Th. ^230^Th and ^210^Pb concentrations also increase until approximately 60 years, then there is a leveling off until 100 years. Reasons for this trend can be explained in terms of half-lives of the radionuclides. Short-lived radionuclides such as ^234^Th (t_1/2_ = 24 days) and ^210^Po (t_1/2_ = 138 days) do not stay for long in the air as they decay, hence very little concentration with time as seen on the graphs. The steady and steep increase from 30 years onwards, shown by ^226^Ra and ^232^Th, can be attributed to their longer half-lives and continual release from sources such as soil or mining activities. Their accumulation may show ongoing exposure from surrounding materials, leading to increased concentrations in the air. These long-lived radionuclides contribute to the human dose at longer timescales [14]. When the concentration within each compartment remains fairly constant in time, this situation is possible for chronic exposure to steady contaminant levels [22]. The initial stability of ^234^Th and ^210^Po could indicate a balance between the sources and sinks of the radionuclides in the air; then the decrease over time suggests that, as they decay, there may be limited resuspension or replenishment from soil or other sources, as these radionuclides are short-lived.

#### Environmental Health Implications for Mining Communities

The results from this study using Ecolego simulation software revealed elevated levels of doses through inhalation attributed to ^234^Th in both adults and infants, whereas ^226^Ra contributed the highest doses to external doses in adults in South African mining communities, posing risks to vulnerable populations. Inhalation of contaminated particles from mining operations may result in serious health consequences such as cancer, reducing mental and nervous system functioning, damaging vital organs, and causing DNA damage [31]. Study area A had the highest total dose, followed by Mine B and Mine C. This shows that community members near Mine A should take precautions to minimize exposure. Inside the body, radium concentrates in the bones, and it decays through gamma radiation to produce radon gas, which can cause cancer [22]. In a recent study in a quarry mining community in rural South Africa, it was reported that the negative effects of the quarry on the environment have always worried the local people. The operations had detrimental effects on human health and safety as well as the environment. It was noted that the company’s disrespect of the community and ignorance of the laws governing quarry mining activities was causing an ethical dilemma [32]. Although regulations have been implemented since 1990, research has shown that the concentration of radionuclides are still high in different ecosystems such as groundwater bodies [33]. For policymakers, there is a need for stricter regulations on mining activities and enforcing air quality monitoring, setting dose limits, or implementing community screenings. Addressing exposure risks can contribute to Sustainable Development Goal number 3 and reduce the burden on the healthcare system. Although direct community consultation was not part of the modeling phase, outreach and educational engagement activities are planned in collaboration with stakeholders such as the National Nuclear Regulator of South Africa to share the findings and promote environmental safety.

It is possible that some results have been overestimated or underestimated, for example, total doses due to inhalation. This can be due to a lack of data or model uncertainties inherent in the estimation of parameter values. Some factors, such as deposition density of radionuclides, have not been considered in the model. For sensitivity and uncertainty analysis, key parameters were varied, such as radionuclide activity concentration and breathing rate, to explore the interactions, as well as running the model under different sets of sampled inputs.

## 5. Conclusions

This study is essential for understanding and mitigating the health risks associated with mining activities, ultimately leading to better health outcomes and community well-being. The results confirm that the dominant contribution to doses was from the ^238^U decay series, with ^226^Ra, a daughter product, contributing a higher dose to the external dose compared with other radionuclides. The total external dose was highest in area A (27 µSv/y), followed by area B (4.63 µSv/y), and the lowest was observed in area C (3.17 µSv/y). Doses due to inhalation in all three study areas, A to C, were higher than in infants. ^234^Th had the highest contribution to doses due to inhalation, and ^210^Po contributed the least doses due to inhalation for the time period of 100 years evaluated. The reason for ^234^Th having the highest contribution was that the ^234^Th concentration in air was set at a higher value (default value of 1.0 × 10^0^ Bq/m^3^) than other radionuclides, hence this is expected. The highest total dose from inhalation of the eight radionuclides for an adult in the study areas was approximately three times higher than the maximum dose for an infant in the study areas. Even if area C had total doses due to inhalation below the world average, it should be known that there is no safe dose for radiation, although it is well known that the higher the dose, the higher the risk of damage. ^234^Th and ^210^Po concentrations in air decrease steadily and approach zero over the 100 years simulated because of removal by decay is which is faster as these radionuclides are short-lived. In mining areas, residents may inhale dust or particulates that settle on food, water, or surfaces, leading to potential ingestion. Hence, future work should look at other pathways, such as contamination from food through ingestion, which were not part of this study, to elucidate the actual total doses that an individual is exposed to.

## Figures and Tables

**Table 1:** Conceptual model presented as an interaction matrix.

source	deposition		
	surface soil	transfer	
		air	transfer
			human

**Table 2:** Inhalation doses in the study areas (µSv/y).

	Radionuclide							
Area	Pb-210	Po-210	Ra-226	Th-230	Th-232	Th-234	U-234	U-238
Adult								
Mine A	0.0466	0.0358	0.0832	0.0613	0.1095	67.45	0.0823	0.0701
Mine B	0.0466	0.0358	0.0832	0.0613	0.1095	271.56	0.0823	0.0701
Mine C	0.0466	0.0358	0.0832	0.0613	0.1095	271.56	0.0823	0.0701
Infant								
Mine A	0.0240	0.0186	0.0406	0.0245	0.0350	43.45	0.0406	0.0350
Mine B	0.0240	0.0186	0.0406	0.0245	0.0350	43.45	0.0406	0.0350
Mine C	0.0240	0.0186	0.0406	0.0245	0.0350	43.45	0.0406	0.0350

## Data Availability

Data are contained within the article.

## Abbreviations

The following abbreviations are used in this manuscript:

ICRP	International Commission on Radiological Protection
AMAD	Activity Median Aerodynamic Diameter

## Appendix A. Input Data for ECOLEGO Simulation

**Table A1:** Radionuclides and their half-lives.

Radionuclide	Half-Life
^238^U	4.5 × 10^9^ years
^234^Th	24 days
^234^U	2.5 × 10^5^ years
^230^Th	7.5 × 10^4^ years
^226^Ra	1600 years
^210^Pb	22.2 years
^210^Po	138 days
^232^Th	1.405 × 10^10^ years

**Table A2:** Committed Effective Dose Coefficients for Inhalation (Sv/Bq) [12,17].

Nuclide	Adult	Infant
^238^U	8.0 × 10^−6^	2.5 × 10^−5^
^232^Th	2.5 × 10^−5^	5.0 × 10^−5^
^210^Pb	5.6 × 10^−6^	1.8 × 10^−5^
^226^Ra	9.5 × 10^−6^	2.9 × 10^−5^
^210^Po	4.3 × 10^−6^	1.4 × 10^−5^
^230^Th	1.4 × 10^−5^	3.5 × 10^−5^
^234^Th	7.7 × 10^−9^	3.1 × 10^−8^
^234^U	9.4 × 10^−6^	2.9 × 10^−5^

The slow (S) retention causes the highest exposure for most radionuclides except for isotopes of actinides such as Np, Pu, Am, and Cm, and that is the reason for using the S values in this study [11].

**Table A3:** Inhalation rate.

Adult	Infant
1 m^3^/h	0.16 m^3^/h

**Table A4:** Recommended dose coefficients (Sv/h per Bq/m^3^) for external exposure [11,17].

Nuclide	${dosCoef}_{ext}$
^238^U	1.5 × 10^−18^
^232^Th	8.8 × 10^−18^
^226^Ra	5.6 × 10^−16^
^210^Pb	3.8 × 10^−17^
^210^Po	9.5 × 10^−19^
^230^Th	2.1 × 10^−17^
^234^Th	4.12 × 10^−16^
^234^U	6.6 × 10^−18^

NB: The values are derived assuming homogeneous distribution in soil.

**Table A5:** Concentration of radionuclides in air.

Nuclide	${Concentration}_{air}$ (Bq/m^3^)
^238^U	1.0 × 10^−6^
^232^Th	5.0 × 10^−7^
^226^Ra	1.0 × 10^−6^
^210^Pb	9.5 × 10^−7^
^210^Po	9.5 × 10^−7^
^230^Th	5.0 × 10^−7^
^234^Th	1.0 × 10^0^
^234^U	1.0 × 10^−6^

**Table A6:** Concentration of radionuclides in soil (Bq/kgDW).

Nuclide	A	B	C
^238^U	151.76	73.39	15.20
^232^Th	47.52	27.29	6.62
^226^Ra	147	113.14	53.59
^210^Pb	151	72	15
^210^Po	150	70	14.5
^230^Th	44.5	25	6.00
^234^Th	40	23	6.5
^234^U	140.5	65.5	12.0

NB: DW means Dry Weight.

**Table A7:** Concentration in dust kgDW/m^3^.

Value	3.13 × 10^−5^
PDF	norm (mean = 2.1 × 10^−5^, sd = 3.38 × 10^−7^, trmin = 6.618 × 10^−6^, trmax = 5.63 × 10^−5^)
Min value	6.616 × 10^−6^
Max value	5.63 × 10^−5^

**Table A8:** Leaching rate.

Nuclide	Leach Rate
^238^U	0.02
^232^Th	0.005
^226^Ra	0.005
^210^Pb	0.03
^210^Po	0.04
^230^Th	0.05
^234^Th	0.05
^234^U	0.025
